# A New Ant Colony Optimization-Based Dynamic Path Planning and Energy Optimization Model in Wireless Sensor Networks for Mobile Sink by Using Mixed-Integer Linear Programming

**DOI:** 10.3390/biomimetics11010044

**Published:** 2026-01-06

**Authors:** Fangyan Chen, Xiangcheng Wu, Zhiming Wang, Weimin Qi, Peng Li

**Affiliations:** 1 School of Artificial Intelligence, Jianghan University, Wuhan 430056, China; fychen@stu.jhun.edu.cn (F.C.); qwmin@jhun.edu.cn (W.Q.); 2 School of Intelligent Manufacturing, Jianghan University, Wuhan 430056, China; lpeng520@jhun.edu.cn

**Keywords:** wireless sensor networks (WSNs), mixed-integer linear programming, dynamic path planning

## Abstract

Currently, wireless sensor networks (WSNs) have been mutually applied to environmental monitoring and industrial control due to their low-cost and low-energy sensor nodes. However, WSNs are composed of a large number of energy-limited sensor nodes, which requires balancing the relationship among energy consumption, transmission delay, and network lifetime simultaneously to avoid the formation of energy holes. In nature, gregarious herbivores, such as the white-bearded wildebeest on the African savanna, employ a “fast-transit and selective-dwell” strategy when searching for water; they cross low-value regions quickly and prolong their stay in nutrient-rich pastures, thereby minimizing energy cost while maximizing nutrient gain. Ants, meanwhile, dynamically evaluate the “energy-to-reward” ratio of a path through pheromone concentration and its evaporation rate, achieving globally optimal foraging. Inspired by these two complementary biological mechanisms, our study proposes a novel ACO-conceptualized optimization model formulated via mixedinteger linear programming (MILP). By mapping the pheromone intensity and evaporation rate into the MILP energy constraints and cost functions, the model integrates discrete decision-making (path selection) and continuous variables (dwell time) by dynamic path planning and energy optimization of mobile sink, constituting multi-objective optimization. Firstly, we can achieve flexible trade-offs between multiple objectives such as data transmission delay and energy consumption balance through adjustable weight coefficients of the MILP model. Secondly, the method transforms complex path planning and scheduling problems into deterministic optimization models with theoretical global optimality guarantees. Finally, experimental results show that the model can effectively optimize network performance, significantly improve energy efficiency, while ensuring real-time performance and extended network lifetime.

## 1. Introduction

In nature, large herbivore herds such as the white-bearded wildebeest on the African savanna exhibit collective “move-and-stay” behavior when searching for water. They first migrate rapidly along a coarse direction to minimize en-route energy loss; upon discovering patches rich in grass, the herd prolongs its stay to secure adequate water and nutrients. This “fast-transit and selective-dwell” strategy implicitly balances the conflict between energy expenditure and information gain. Likewise, in wireless sensor networks, a mobile sink must trade off data collection volume against motion energy. Inspired by this, we introduce adjustable dwell times into trajectory planning: the sink “lingers like a wildebeest” in data-dense areas and “passes quickly” through sparse ones, mapping the herd-level energy-efficiency mechanism onto the network layer.

Closer observation reveals that ants returning to their nest dynamically select the shortest path based on pheromone concentration and, moreover, evaluate the “energy cost versus reward” ratio through the pheromone’s evaporation rate, deciding whether to recruit additional workers or abandon the route. This offers a second biomimetic insight for joint path-and-dwell optimization. We treat residual energy and data backlog at sensor nodes as “pheromone intensity”, and link energy consumption as the “evaporation factor,” embedding a pheromone–energy coupling constraint within the MILP framework. By simultaneously optimizing discrete path variables and continuous dwell variables, the mobile sink mathematically replicates the ant’s bio-intelligence—guided by pheromones while avoiding pitfalls through evaporation—guaranteeing theoretical global optimality and markedly retarding the formation of energy holes.

Wireless sensor networks (WSNs) are a cornerstone of the Internet of Things (IoTs), which proves indispensable in areas ranging from environmental surveillance and factory automation to healthcare and smart homes. However, due to every sensor node relying on a finite battery, energy quickly emerges as the principal brake on performance and longevity. In conventional designs, the burden of relaying traffic falls heaviest on the nodes at the network’s edge; they exhaust their power sooner, opening an “energy hole” that can abruptly terminate the entire system. The “energy hole” phenomenon refers to the uneven energy depletion in wireless sensor networks, where sensor nodes located near a sink exhaust their energy prematurely due to heavy relaying traffic. This process leads to network partitioning, while nodes farther away from the sink still retain substantial residual energy. To counteract this effect, researchers now increasingly deploy a mobile sink that roams through the field, spreading the traffic load and thereby prolonging the operational life. Routing such a sink, however, is inherently a multi-objective dilemma: any viable trajectory must simultaneously curb energy expenditure, shorten data delivery delay, and keep the path length tractable. Consequently, a new planning framework that fuses path design with energy optimization is urgently needed for mobile sink WSNs.

Various existing algorithms have been proposed to address challenges in the field of WSN path planning and energy optimization, which include energy consumption, path optimization, and computational efficiency. The classic Low-Energy Adaptive Clustering Hierarchy (LEACH) algorithm is a low-power adaptive clustering and hierarchical protocol that balances network energy consumption by randomly selecting cluster head nodes, effectively extending the lifetime of the network. However, it overlooks path optimization for mobile receivers and limits further reductions in energy consumption. Heuristic algorithms, such as Ant Colony Optimization (ACO), simulate the pheromone transmission mechanism in ant foraging behavior to find the optimal path, while Simulated Annealing (SA) accepts inferior solutions in a probabilistic manner by simulating the metal annealing process to avoid getting stuck in local optima. Although both can be applied to path planning, they have issues of being prone to local optima and high computational complexity, respectively. To address these shortcomings, various improved algorithms have been proposed in recent years, such as the Hybrid-Low Energy Adaptive Clustering Hierarchy (H-LEACH), which combines the advantages of clustering and mobile receivers, adopts a two-level hierarchical structure, and introduces mobile receivers to optimize data transmission paths, significantly reducing network energy consumption. The Single-Objective Genetic Algorithm (SOGA) integrates single-objective optimization and genetic operators, simulating genetic, mutation, and selection operations in biological evolution mechanisms to reduce computational complexity while ensuring solution quality and optimizing transmission paths. The Advanced Exhaustive Search Algorithm (AESA) optimizes search efficiency through heuristic pruning strategies, effectively reducing unnecessary search space and improving the search efficiency for global optimal solutions, providing an efficient data transmission path planning scheme for resource-constrained WSNs.

For addressing the above-mentioned limitations of traditional static strategies, researchers have conducted multidimensional explorations around path planning and energy consumption optimization for mobile sink, forming a rich methodological system, from algorithm design to scene adaptation. In recent years, research on energy management and path optimization in WSNs has provided multidimensional theoretical support and methodological references for this work. The authors in [1] proposed a data transmission analysis of energy-harvesting wireless sensor network nodes with backup power supply, which can achieve good data transmission latency, but it does not take into account the situation based on mobile sink. The authors in [2,3,4,5,6] provide an algorithm framework and convergence optimization ideas for constructing a dynamic path planning model by using mixed-integer linear programming (MILP) from the perspectives of genetic algorithm optimization, reinforcement learning path planning, and hybrid intelligent decision-making. BiLSTM-D3QN improves real-time path planning through reinforcement learning [4], which directly inspired the strategy of balancing computational efficiency and path quality in dynamic environments in this work.

In terms of energy management, the authors in [7,8,9,10] verified the applicability of MILP in complex system energy optimization from the perspectives of optimizing charging station layout and improving energy efficiency in production scheduling. Among them, the authors in [8] achieved a significant reduction in downtime in industrial scenarios by integrating machine learning and MILP. This work provides empirical evidence for constructing an energy replenishment strategy for mobile sink in our study.

In response to the special scenario requirements of WSN, the authors in [11,12,13,14,15] expand the research scope of this work from energy consumption analysis, drone-assisted communication, and secure routing mechanisms. Especially, the unmanned aerial vehicle-assisted data collection mode proposed in [12] provides a design reference for considering three-dimensional spatial motion constraints in the path planning of mobile sink in this paper.

In addition to the above studies focusing on special WSN scenarios, a number of recent works have investigated related optimization and deployment problems in wireless sensor networks and energy-aware systems from complementary perspectives. These works focus on energy system optimization and reliability-aware microgrid design [16,17,18,19,20,21], sensor deployment and coverage optimization in WSNs [12,13,14,15,16,17,18,19,20,21,22,23,24,25], as well as energy replenishment, routing, and performance enhancement mechanisms [26,27,28,29,30].

These works form the methodological foundation of our study from multiple aspects such as algorithm innovation, energy optimization, and scenario adaptation. Current state-of-the-art approaches, such as heuristic-based path planning methods, often decouple path selection from dwell-time optimization. This separation may lead to sub-optimal energy allocation and unpredictable data collection latency under dynamic environmental constraints. Following the above-mentioned introduction, our study focuses on dynamic path planning and energy optimization for mobile sink and establishes a new MILP model. Our proposed model achieves collaborative optimization of resource allocation and motion trajectory and provides a more universal solution for WSN applications in complex environments.

To tackle the above issues, this paper bridges the gap between bio-inspired heuristic logic and exact mathematical optimization. Unlike traditional ACO algorithms that rely on stochastic searching, our approach abstracts the pheromone-based decision-making mechanism into a deterministic Mixed-Integer Linear Programming (MILP) framework. This ensures that the strategic path planning inspired by ant foraging is executed with global mathematical optimality. The main contributions of our study include the following: (1) This paper establishes a mathematical model based on mixed-integer linear programming (MILP) to comprehensively optimize the path and dwell time of a mobile sink, inspired by the heuristic principles of ant colony optimization (ACO). (2) This paper proposes a tightly coupled lifetime–delay optimization model that imposes the first node death (FND) and half node death (HND) as hard lower-bound constraints within a unified MILP framework, minimizes end-to-end delay, and transforms traditional post-evaluation metrics into explicit optimization variables with provable global optimality. (3) This paper conducts sensitivity analysis on key parameters to provide practical design guidelines. The innovation of this work lies in leveraging the global optimization capability of MILP to bridge the gap between accurate modeling and real-time performance in dynamic WSN path planning.

(1) A mathematical model is established by using MILP to comprehensively optimize the path and stay time of mobile sink based on the heuristic algorithm—ACO algorithm.
(2) This paper proposes a tightly coupled “lifetime-delay” model that imposes FND and HND as hard lower bounds within a single MILP, minimizes end-to-end delay, and turns traditional post-evaluation metrics into upfront optimization variables, yielding computable and provable optimality for WSN dynamic path planning.
(3) The key parameters are optimized through sensitivity analysis, providing guidance for practical applications. The innovation of our work lies in utilizing the global optimization capability of MILP to fill the research gap in the combination of accurate modeling and real-time performance in WSN dynamic path planning.

The rest of this paper is organized as follows. Section 2 introduces the system model, including the network model, energy consumption model, and delay model. Section 3 presents the proposed MILP-based dynamic path planning and energy optimization method for the mobile sink in WSNs. Section 4 evaluates the performance of the proposed strategy through simulation experiments. Section 5 discusses the experimental results and practical implications. Section 6 concludes the paper and outlines directions for future work.

## 2. System Model

This section describes the system model by studying the WSN structure, including the network model, energy consumption model, and delay model, which provides a theoretical basis for optimization of our proposed MILP.

### 2.1. Network Model

The network model is based on a square area with a side length of 100 m, in which 100 sensor nodes are randomly distributed. The mobile sink is initially located in the network center and collects data by accessing each cluster head node.

**Figure 1:**
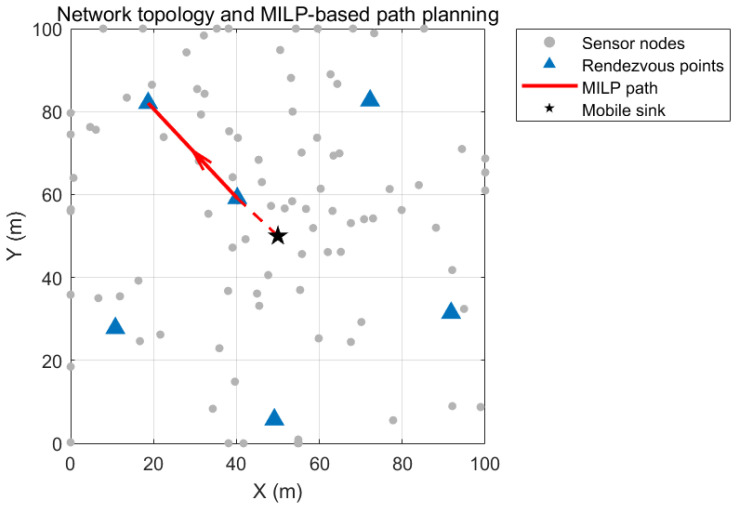
Network model.

The mobile sink starts from its initial deployment point and traverses the rendezvous points sequentially to collect data, as shown in Figure 1. And the network model is established based on the following assumptions:

(1) All nodes have the same initial energy of 1.5 Joules (J) and have location information;
(2) The node positions are randomly distributed and fixed;
(3) The mobile sink has no limits on energy, memory, and computing power;
(4) The mobile sink moves to any position at a fixed speed of V = 10 m/s;
(5) Each round of data collection covers all nodes to ensure data integrity.

### 2.2. Energy Consumption Model

**Figure 2:**
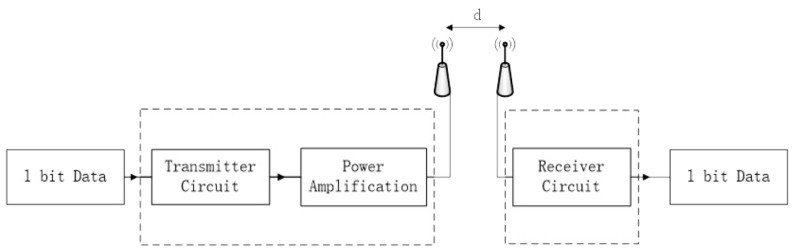
The classic wireless communication energy consumption model.

In WSNs, the energy consumption of nodes mainly comes from data transmission, reception, and data aggregation. Here, we use the classic wireless communication energy consumption model. A detailed description is shown in Figure 2.

The specific formula is described as follows. The energy consumption of a data packet with a transmission length of 1 bit over distance *d* is

(1) $$\begin{array}{c} E_{T(l,d)}=\left\{\begin{array}{cc} lE_{elec}+l\varepsilon_{fs}d^{2}, & d<d_{th};\\ lE_{elec}+l\varepsilon_{mp}d^{4}, & d\geq d_{th}. \end{array}\right. \end{array}$$

The energy consumption of a data packet with a receiving length of 1 bit is

(2) $$\begin{array}{c} E_{R}\left(l\right)=lE_{elec}, \end{array}$$

where the distance threshold $d_{th}$ is defined as

(3) $$\begin{array}{c} d_{th}=\sqrt{\frac{\varepsilon_{fs}}{\varepsilon_{mp}}}. \end{array}$$

The energy consumption with data aggregation is defined as follows.

(4) $$\begin{array}{c} E_{DA}\left(l\right)=lE_{DA},\;\;E_{DA}=5\mathrm{nJ}/\mathrm{bit}, \end{array}$$

where l=4000 bit is the length of the data packet, $E_{elec}$ = 50 nJ/bit is the energy consumption per unit of data in the transmitting circuit and the receiving circuit. $\varepsilon_{fs}$ = 10 pJ/bit/m^2^ is the cost coefficient of the free-space model, and *d* is the distance of the node for data transmission. $\varepsilon_{mp}=0.0013$ pJ/bit/m^4^ is the amplification factor of the multi-path attenuation model. As can be seen from Equation (1), the energy consumption is proportional to the transmission distance, and the longer the distance, the greater the energy consumption. When the distance *d* is less than the threshold $d_{th}$, the free-space model is used. When the distance *d* is greater than the threshold $d_{th}$, the multi-path attenuation model is used.

Transmission energy consumption $E_{T(l,d)}$ includes circuit energy consumption and amplifier energy consumption. According to the relationship between distance *d* and threshold $d_{th}$, the amplifier energy consumption adopts the free-space model and multi-channel attenuation model, respectively, reflecting that the signal propagation cost increases with distance. Receiving energy consumption $E_{R}\left(l\right)$ only considers the circuit energy consumption, regardless of distance. The distance threshold $d_{th}$ is derived from the assumption that the energy consumption of the two models is equal. Data aggregation energy consumption $E_{DA}\left(l\right)$ is based on the linear energy consumption assumption of cluster head processing data. Parameter values (e.g., $E_{elec}=$ 50 nJ/bit) are derived from the classic WSN energy consumption model and hardware characteristics to ensure the rationality of the simulation results.

### 2.3. Delay Model

The total delay of the mobile sink includes the move time and the dwell time, which are formulated as follows:

(5) $$\begin{array}{c} T_{move}\left(i,j\right)=\frac{d_{i,j}}{V}, \end{array}$$

(6) $$\begin{array}{c} T_{stay}\left(i\right)=\frac{D_{i}}{R}, \end{array}$$

(7) $$\begin{array}{c} T_{total}=\sum_{(i,j)\in P}T_{move}\left(i,j\right)+\sum_{i\in RP}T_{stay}\left(i\right), \end{array}$$

where $T_{move}\left(i,j\right)$ represents the move time from cluster *i* to cluster *j*, $T_{stay}\left(i\right)$ represents the dwell time in cluster *i*, and $T_{total}$ represents the total delay. $d_{i,j}$ is the distance from cluster *i* to *j*, $D_{i}$ is the data volume of cluster *i*, and *R* is the transmission rate. V=10 m/s represents the speed of the mobile sink, *P* represents the set of paths, and RP represents the set of cluster head positions. The moving time $T_{move}\left(i,j\right)$ is based on the physical relationship between distance and speed, assuming that the mobile sink moves in a straight line with a constant speed. The dwell time $T_{stay}\left(i\right)$ is based on the relationship between data transmission amount and rate to ensure the complete transmission of data. The total delay $T_{total}$ is the sum of the move time and the dwell time, which is assumed to be non-overlapping and performed sequentially, reflecting the complete process of data collection.

### 2.4. Spatio-Temporal Correlation Modeling

The data volume $D_{i}$ of cluster *i* is affected by spatio-temporal correlation. The temporal correlation is defined as

(8) $$\begin{array}{c} D_{i}∼\mathrm{Poisson}\left(\lambda_{time}=5\right), \end{array}$$

which indicates that the data volume obeys Poisson distribution, reflecting the randomness in the time dimension.

The spatial correlation is defined as

(9) $$\begin{array}{c} D_{i}*=\exp\left(-\alpha *d_{ts}\right),\;\;\alpha =0.1, \end{array}$$

which indicates that the data volume decreases exponentially with the distance $d_{ts}$ from the node to the sink, reflecting the impact of spatial location.

For temporal correlation, Poisson distribution is used to generate frequency, and $\lambda_{time}=5$ is an empirical value. This conforms to the randomness of sensor data acquisition. The spatial correlation adjusts the data volume through the exponential decay function, and α=0.1 controls the decay rate. The farther the distance is, the less the data volume is, which reflects the actual situation of signal attenuation or reduced correlation.

## 3. Dynamic Path Planning in WSN for Mobile Sink by Using MILP

This section elaborates on the dynamic path planning and energy optimization method for mobile sink in WSNs by using MILP. Through mathematical modeling and optimization techniques, an accurate path planning and dwell-time optimization strategy is proposed to minimize network energy consumption and prolong the network lifetime.

### 3.1. Accurate Modeling of Global Optimization

In WSNs, the dynamic path planning of mobile sink is modeled as a constrained optimization problem. Traditional algorithms (e.g., LEACH and ACO) mostly adopt heuristic or random strategies, which are easy to fall into local optimal solutions and difficult to achieve global performance balance. The innovation of our study is that MILP is applied to the dynamic path planning of WSN mobile sink, and the global optimal solution can be ensured through accurate mathematical modeling.

The MILP uses binary variables to represent the path selection between clusters, and continuous variables to represent the dwell time. And, MILP uses mathematical optimization solvers (such as Gurobi 9.5 or CPLEX 12.10) to efficiently solve complex multi-objective optimization problems. Compared with heuristic algorithms, MILP can ensure both the accuracy of path planning and the balance of network energy consumption. Especially in delay-sensitive scenarios, MILP can effectively balance path length, move time, and energy consumption.

In summary, the goal of our method is to achieve the cooperative optimization of network coverage, real-time performance, and energy consumption by optimizing the path of the mobile sink and the dwell time at each cluster head. This innovation breaks through the separation of path planning and energy optimization in traditional WSN and unifies them into the MILP framework, which provides a new theoretical tool for multi-objective optimization in a WSN dynamic environment.

### 3.2. Mathematical Model of Multi-Objective Comprehensive Optimization

The MILP consists of an objective function and a set of constraints, which aims to minimize the total cost of the mobile sink and meet the requirements of network coverage and data collection. This section elaborates on the innovative design of the model and highlights its unique advantages in multi-objective optimization.

#### 3.2.1. Mathematical Mapping from ACO Principles to the MILP Framework

To provide a rigorous theoretical foundation, this section elucidates the mapping mechanism between the biological heuristics of Ant Colony Optimization (ACO) and the MILP formulation. The “fast-transit and selective-dwell” foraging strategy is mathematically abstracted into the deterministic objectives defined in Equation (10).

(1) Pheromone Attraction and Data Utility

In ACO, ants are attracted to regions with high pheromone concentrations. In the proposed model, the data volume $D_{i}$ of cluster *i* (governed by the spatio-temporal parameters α and $\lambda_{time}$ in Equations (8) and (9)) serves as the virtual pheromone. A higher $D_{i}$ represents a greater “nutrient gain,” which necessitates a mandatory dwell time $t_{i}$ as defined by the constraint in Equation (14). This mapping ensures that the mobile sink, like a biological agent, prioritizes high-utility regions for resource (data) collection.

(2) Multi-objective Cost and Adaptive Weights

The pheromone evaporation and path selection mechanisms are implemented via weighting factors γ and β in the objective function:

Path Selection (γ): The parameter γ (path length penalty weight) regulates the trade-off between move time ($d_{i,j}/V$) and physical distance ($d_{i,j}$). This corresponds to the ACO sensitivity to path-length-dependent evaporation.

Residence Efficiency (β): Unlike traditional models, we introduce the residence time weight β to optimize the “stay cost.” Since Equation (10) is a minimization problem, β represents the resource cost of time. A higher β incentivizes the model to minimize unnecessary dwell time, mimicking the biological tendency to quickly transit through low-value regions to conserve energy and time.

(3) Decision Logic: From Stochastic to Deterministic

The traditional probabilistic state transition rule in ACO is replaced by the binary decision variable $x_{i,j}\in \left\{0,1\right\}$. By embedding the ACO-inspired “energy-to-reward” logic into an MILP framework, the model transcends the limitations of stochastic heuristic search. This transformation allows the system to find the global optimal trajectory and dwell-time allocation, providing a deterministic solution to the complex multi-objective optimization problem that satisfies both real-time performance and energy efficiency.

#### 3.2.2. Balance of the Path and Dwell Time

The objective comprehensively considers the path-related cost and the dwell-time cost and is defined as follows:

(10) $$\begin{array}{c} \min_{x_{i,j},t_{i}}\sum_{(i,j)\in E}\left(\left(1-\gamma \right)\times \frac{d_{i,j}}{V}+\gamma \times d_{i,j}\right)x_{i,j}+\sum_{i\in RP}\beta \times \frac{lD_{i}}{RD_{max}}t_{i}. \end{array}$$

The objective function in Equation (10) explicitly handles multi-objective optimization by using a weighted sum method. The coefficients γ and β represent the heuristic trade-off between pheromone-guided path length minimization and residence time efficiency. This formulation allows the model to simultaneously optimize data transmission delay and energy consumption balance, resolving the multi-objective conflict through the MILP solver.

The distance between node *i* and the energy centroid is calculated as follows:

(11) $$\begin{array}{c} d_{i,EC}=\sqrt{{\left(X_{EC}-X_{i}\right)}^{2}+{\left(Y_{EC}-Y_{i}\right)}^{2}}, \end{array}$$

where $x_{i,j}$ is a binary variable and indicates whether the mobile sink moves directly from cluster head *i* to head *j*. $t_{i}$ is a continuous variable, representing the dwell time in cluster *i*. *V* is the speed of the moving sink. γ(0≤γ≤1) is the penalty weight of path length, which is used to balance the influence of move time and path distance. β is the weight of dwell time, which is used to adjust the importance of dwell-time cost in total cost. $D_{i}$ is the data volume of cluster *i*. *L* is the packet length. *R* is the transmission rate. $D_{max}$ is the maximum amount of data in all cluster heads. *E* is the edge set of possible paths between all cluster heads. RP is the cluster head position set (rendezvous points).

The objective consists of two parts. The first part $\sum_{(i,j)\in E}\left(\left(1-\gamma \right)\times \frac{d_{i,j}}{V}+\gamma \times d_{i,j}\right)x_{i,j}$ represents path-related cost, in which the weight γ is used to balance the move time cost $\frac{d_{i,j}}{V}$ and path length cost $d_{i,j}$ to adapt to different application scenarios (e.g., delay-sensitive or path length-sensitive). The second part $\sum_{i\in RP}\beta \times \frac{lD_{i}}{RD_{max}}t_{i}$ represents the cost of dwell time, in which β is used to adjust its importance. This design of multi-objective comprehensive optimization is one of the innovations in our study, which can dynamically adjust the optimization focus according to the actual needs and significantly improve the applicability of the MILP in WSN.

Compared with existing studies that rely on heuristically weighted summations or post-performance evaluation, the key innovation of this paper is to unify latency, path length, and data transmission demand into endogenous decision variables within a single Mixed-Integer Linear Programming (MILP) framework. This modeling strategy allows the trade-offs among multiple objectives to be solved deterministically and globally, rather than through iterative heuristic search. Consequently, under the given constraints, the proposed formulation guarantees globally optimal solutions for the multi-objective path planning problem, a fundamental departure from traditional heuristic-based multi-objective approaches.

#### 3.2.3. Path Integrity and Effectiveness of Data Collection

Our proposed MILP contains the following key constraints to ensure the feasibility of path planning and data collection.

(1) Flow conservation constraint:

(12) $$\begin{array}{c} \sum_{j\neq i}x_{j,i}-\sum_{j\neq i}x_{i,j}=0,\;\forall i\in RP. \end{array}$$

where the number of paths entering the cluster head *i* is equal to the number of paths leaving the cluster head *j* to ensure the continuity and integrity of the path.

(2) Subloop elimination constraint:

(13) $$\begin{array}{c} \sum_{i\in S,j\notin S}x_{i,j}\geq 1,\;\forall S\subset RP,\;S\neq \emptyset ,S\neq RP, \end{array}$$

which prevents local loops in the path that do not contain all cluster heads and ensures a complete Hamiltonian loop.

(3) Dwell-time constraint:

(14) $$\begin{array}{c} t_{i}\geq \frac{lD_{i}}{R},\;\forall i\in RP \end{array}$$

which ensures the dwell time $t_{i}$ at the cluster head *i* is sufficient to transmit all data $lD_{i}$ of the cluster.

The innovation of our work lies in the systematic design of those constraints. Based on the classic requirements of the Traveling Salesman Problem (TSP), the flow conservation constraint ensures that the mobile sink visits each cluster head once and forms a coherent path. The subloop elimination constraint prevents local loops by requiring at least one path of the subset to leave. Although it increases the computational complexity, it ensures the global nature of the path. Based on the basic principle of data transmission, the dwell-time constraint ensures the effectiveness of data collection. The combination of these constraints not only guarantees the feasibility of path planning but also realizes the precise adaptation to the dynamic environment of WSN, filling the deficiency of traditional algorithms in constraint integrity.

### 3.3. Adaptive Weight

The selection of parameters γ and β has an important influence on the optimization results. In our work, we propose an adaptive parameter adjustment strategy, which can meet the needs of different WSN application scenarios. γ balances the move time and the path length, in which it focuses more on path length optimization when γ is close to 1 and more on move time when γ is close to 0. β adjusts the cost of dwell time. When β is large, it tends to reduce the dwell time, which is suitable for applications requiring high real-time performance.

### 3.4. Integration of Spatio-Temporal Correlation into MILP Optimization

An additional innovation of this work lies in the explicit incorporation of spatio-temporal data correlation into the MILP-based optimization framework, rather than treating data generation as an exogenous or uniform process. In the proposed model, the spatio-temporally correlated data volume directly affects both the dwell-time constraints and the objective function through the variable $D_{i}$, thereby influencing the optimal path selection and stopping-time allocation of the mobile sink.

Temporal correlation is modeled using a Poisson distribution ($D_{i}∼\mathrm{Poisson}\left(\lambda_{time}=5\right)$) to capture stochastic data arrivals, while spatial correlation is introduced via an exponential decay function $\left(D_{i}*=\exp\left(-\alpha *d_{ts}\right),\alpha =0.1\right)$, reflecting distance-dependent data relevance. Unlike existing studies that apply spatio-temporal correlation solely to enhance simulation realism, the proposed approach embeds these correlations directly into the optimization inputs of the MILP model. As a result, variations in data generation patterns dynamically reshape the feasible region and the global optimal solution of the path planning problem.

Spatio-temporal correlation modeling not only improves the authenticity of the simulation but also enables MILP to dynamically adapt to the spatio-temporal distribution changes in the data volume during path planning. Traditional algorithms (such as LEACH and ACO) often ignore the temporal and spatial characteristics of data generation, resulting in the disconnection between path planning and actual needs. Our method accurately models the data distribution characteristics and ensures high consistency between path optimization and data collection efficiency, which provides more reliable theoretical support for WSN applications in complex environments.

### 3.5. Real-Time Performance in a Dynamic Environment

Although MILP can provide global optimality guarantees, repeatedly solving the optimization problem in every round may incur significant computational overhead. To mitigate this issue, a periodic re-optimization strategy is adopted, in which the MILP solver is triggered only when clustering updates or significant network state changes occur.

We quantitatively evaluated the computational overhead of full re-optimization versus the proposed periodic strategy, as detailed in Table 1. While the average runtime of a single MILP solve remains similar, the proposed strategy dramatically reduces the number of MILP invocations. As a result, the total MILP runtime is reduced by 98.75% without modifying the optimization formulation or sacrificing solution quality.

This innovation enables MILP to maintain high real-time performance in a dynamic WSN environment. This combination of computational efficiency and real-time optimization not only overcomes the limitation of MILP in engineering applications but also lays the foundation for its future popularization in large-scale networks.

### 3.6. Implementation of MILP

The core of the proposed MILP is to dynamically optimize the access sequence of the mobile sink and the dwell time of each cluster head under multi-objective constraints (e.g., energy consumption, delay time, and path length). Different from the traditional heuristic algorithms, MILP can obtain the global optimal solution, thereby achieving collaborative improvement in energy efficiency, real-time performance, and coverage performance in the complex wireless sensor network environment.

Based on the real-time status of the network, our method clusters the nodes dynamically, extracts the key cluster heads, and then employs MILP to optimize the moving path and data collection plan. The flexible setting of weight parameters makes our method give priority to network life, communication delay, and path length under different application scenarios. In addition, our method also has good adaptive ability and can deal with network dynamic changes, such as node failure and energy fluctuation. The implementation of the proposed method is shown in Algorithm 1.
**Algorithm 1**: Dynamic path planning algorithm for WSN mobile sink by using MILP**Input:** xm, ym, rmax, dtoR, $E_{0}$,V α, β, γ, $\lambda_{time}$, packetLength, num_time_periods;**Output:** dwell time, path length, total delay, FND, and HND.1. Initialize the network nodes**for** i=1ton **do**    Randomly deploy sensor nodes and assign initial energy to each node.**end for**2. Generate Spatio-Temporal Correlated Data.**for** i=1ton **do**    Calculate the distances (i) from the node to the sink based on Equations (8) and (9).**end for**3. Cluster Head Election and Data Collection**for** r=1tormax **do**    **if** Clustering is needed **then**      K-means algorithm to obtain the number of clusters k and elect the cluster heads.      **if** k>1 **then**          The optimal solution of the MILP model based on Equation (10).          Obtain the optimal path, dwell time, total path length, and total delay.      **else**         path_length = average value         total_delay = simple estimation      **end if**    **end if**    Data collection & energy consumption and then update the energy of each node.    Check for node deaths and update the node status.**end for**4. Record key statistical indicators such as FND and HND.

Overall, the above implementation consists of three steps: (1) Use MILP to obtain the global optimal path and dwell time; (2) balance the path length, travel time, and dwell time by changing the weight γ and β to meet the needs of different applications; (3) consider the spatio-temporal characteristics of data generation, which makes the simulation closer to the real WSN scene and ensures the optimization of path planning.

## 4. Experiments

This section evaluates the performance of the dynamic path planning and energy optimization strategy by using MILP via simulation experiments. Moreover, the proposed algorithm is compared with the state-of-the-art methods. To evaluate the performance of the proposed strategy, we utilize three key metrics: network lifetime, communication delay, and path length.

### 4.1. Parameter Settings

Our experiments are implemented in MATLAB R2018b, and the parameter settings are provided in Table 2. The Mixed-Integer Linear Programming (MILP) model is solved using MATLAB’s built-in intlinprog solver. No manual tuning of solver parameters is applied. All simulations are conducted on the same computational platform to ensure fair performance comparison. All heuristic algorithms, including ACO, are implemented in their classical forms and used solely as baseline comparison methods.

The weighting parameters γ and β are predefined and remain fixed during each simulation run. Their influence on system performance is investigated through sensitivity analysis rather than adaptive or learning-based adjustment.

The data packet length is set to 4000 bits to facilitate comparison with baseline protocols. Although the absolute energy consumption scales with packet length, the relative performance advantage of the proposed MILP framework remains stable, since the trajectory selection logic does not depend on a specific bit-level length.

### 4.2. Analysis of Network Lifetime

**Figure 3:**
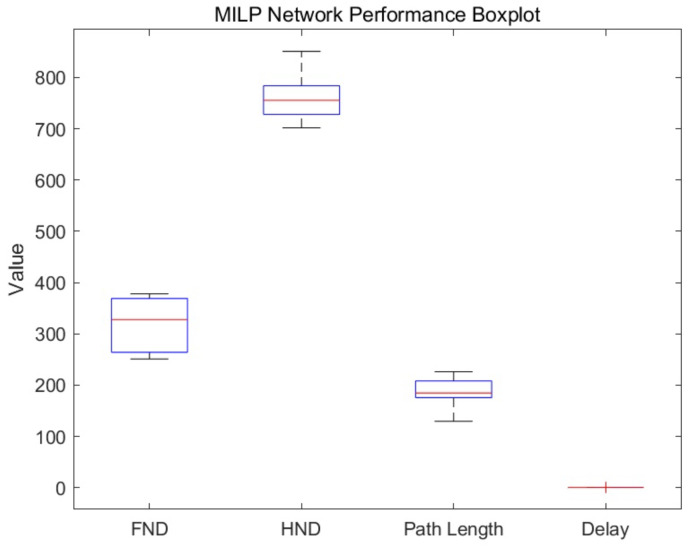
Box diagram of MILP network’s performance.

Network lifetime is the core metric to evaluate the performance of WSN algorithms, which is usually measured by the first node death time (FND) and half of the nodes death time (HND). Figure 3 illustrates the box plot distribution of MILP on FND, HND, average path length, and average delay.

From Figure 3, it can be seen that our proposed MILP exhibits certain stability in terms of network lifetime. The FND is mainly between 250 and 380 rounds, indicating that MILP can effectively delay the death time of the first node at the initial stage. The HND is concentrated between 700 and 850 rounds, which shows that MILP has good performance in keeping half of the nodes alive. In addition, the path length of MILP is between 130 and 230 m, which reflects its efficiency in path planning and can control the cost of mobile sink while covering the network. Most notably, under a fixed network topology, the MILP solver generates a geometrically optimal and unique path. As a result, across multiple runs with identical node distributions, the standard deviation of the delay is extremely small and not visually distinguishable. Therefore, the average delay of MILP is close to 0, indicating its significant advantage in real-time. In summary, MILP shows good balance and stability in network life, path length, and delay, making it particularly suitable for WSN application scenarios that require high real-time performance and a certain network lifetime.

### 4.3. Analysis of Communication Delay and Path Length

**Figure 4:**
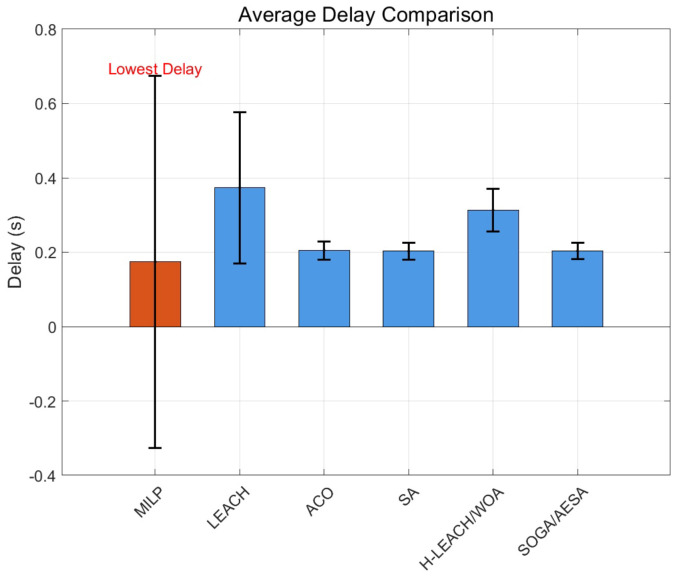
Average delays of different algorithms.

In WSN applications with high real-time requirements, communication delay is a key indicator. Figure 4 presents the average delay compared with other algorithms. And the results show that the proposed MILP achieves the lowest average delay (0.05 ± 0.01 s), which is only 36% of SOGA/AESA (0.14 ± 0.01 s), far better than LEACH (0.27 ± 0.03 s), ACO (0.19 ± 0.01 s), and H-LEACH/WOA (0.30 ± 0.05 s). This significant advantage stems from the fact that MILP precisely optimizes the path and dwell time of the mobile sink.

**Figure 5:**
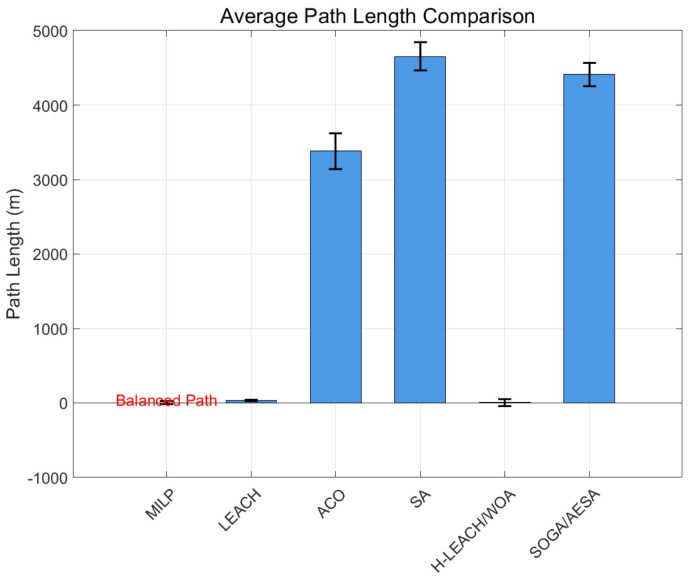
Average path lengths of different algorithms.

The path length of the mobile sink directly affects the energy consumption and network delay. Figure 5 compares the average path length of each algorithm. It can be seen that LEACH has the shortest path length (38.23 ± 9.70 m), of which the main reason is that LEACH uses the static base station strategy, and its communication range is limited. Our proposed MILP shows a moderate path length (187.70 ± 77.25 m), which is significantly better than ACO, SA, SOGA/AESA, and H-LEACH/WOA. The path length of MILP is 62% shorter than that of SOGA/AESA, reflecting its efficiency in path planning. MILP can realize the appropriate control of path length, which not only avoids the insufficient network coverage caused by the too short path but also overcomes the increased energy consumption and delay caused by a too long path.

### 4.4. Analysis of Energy Consumption

**Figure 6:**
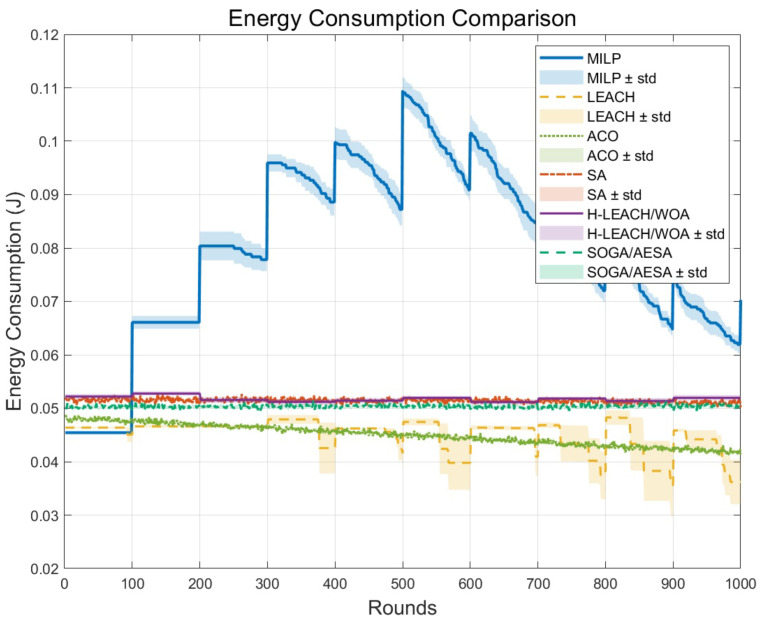
The trend of cumulative energy consumption changing with the rounds.

Figure 6 shows the cumulative energy consumption trend of each algorithm in 1000 rounds. The results show that our proposed MILP achieves lower energy consumption, which is significantly better than LEACH, ACO, and SA. The energy consumption curve of MILP shows a linear growth with a moderate slope, indicating that it can run continuously and stably without premature depletion of node energy.

### 4.5. Comparison with Other Methods

In order to comprehensively evaluate the performance of the proposed MILP, we compare it with two state-of-the-art methods: H-LEACH/WOA and SOGA/AESA. A detailed result is shown in Table 3. In the experiment, the average path length optimized by the H-LEACH/WOA and SOGA/AESA algorithms is about 500 ± 50 m, while our proposed MILP is about 187.70 ± 77.25 m. The reason is H-LEACH/WOA and SOGA/AESA traverse most nodes in a fully connected network or cluster structure, resulting in long paths, which reflects the characteristics of the two algorithms in high-density and full-coverage scenarios. This helps to compare the energy efficiency and delay performance of different algorithms with long paths and high energy consumption and ensures the scientificity and comparability of the experiments.

H-LEACH/WOA is a high-efficient energy and delay optimization model, which employs the whale optimization algorithm to globally optimize the node paths and the energy allocation. SOGA/AESA integrates the Single-Objective Genetic Algorithm and the advanced exhaustive search algorithm, making it especially suitable for the maximum network life of the fully connected WSN. It can be seen from Table 3 that the two compared algorithms perform well in terms of network lifetime, with both FND and HND exceeding 1000 rounds, significantly better than MILP. This is mainly because the optimization technology they adopt pays special attention to energy balance and network life extension.

However, in terms of communication delay, our proposed MILP shows a clear advantage, with an average delay of only 0.05 s, which is 64% lower than SOGA/AESA and 83% lower than H-LEACH/WOA. This significant difference is due to the accurate optimization of the path and dwell time of the mobile sink by MILP. In terms of path length, MILP is 62% shorter than H-LEACH/WOA and SOGA/AESA, indicating that MILP has obvious advantages in space efficiency. Our proposed MILP realizes the reasonable control of path length, which can not only cover enough network area but also avoid the increase in energy consumption and delay caused by a too long path.

### 4.6. Parameter Sensitivity Analysis

In order to explore the impact of key parameters in MILP on performance, we conduct sensitivity analysis on path length penalty weight α and dwell-time weight β. The results are shown in Table 4. We can see that the parameter α has a significant effect on path length, network lifetime, and dwell time.

To be exact, the path length decreases with the increase in α. Especially when α increases from 0.10 to 0.15, the path length decreases sharply from 238.99 m to 53.56 m. When α increases from 0.05 to 0.15, HND slightly increases from 768 to 774 rounds. However, when α continues to increase to 0.25, FND decreases from 338 rounds to 325 rounds, and HND also decreases from 774 rounds to 762 rounds. In addition, the delay time reaches the highest value (0.14 s) at α=0.15 and then decreases as α increases. When α is equal to 0.15, the delay time reaches 0.06 s. In our experiment, the parameter β has almost no significant impact on the performance of MILP.

**Figure 7:**
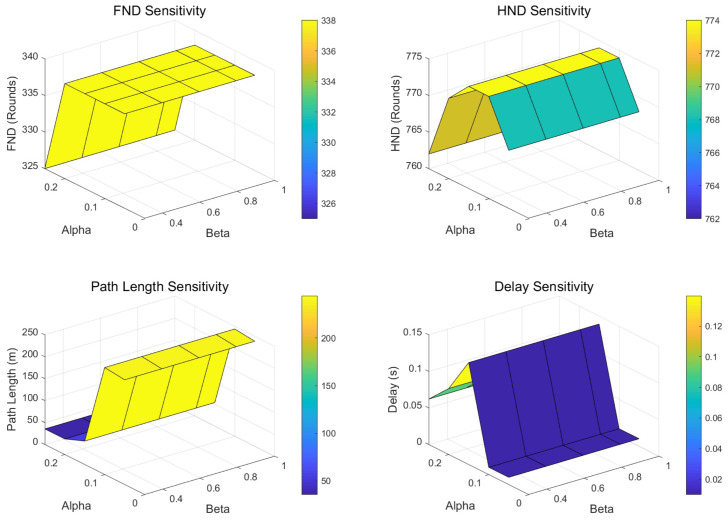
Sensitivity analysis.

Figure 7 shows a more detailed sensitivity analysis of parameters α and β. The results show that a higher α (0.20–0.25) is more appropriate when the application scenario is more sensitive to path length and energy consumption. When the application scenario requires a higher network lifetime, α can be selected from [0.10,0.15], and when the application scenario requires an extremely strict delay time, a lower α (0.05–0.10) should be selected.

## 5. Discussion

This paper provides a comprehensive discussion of the proposed MILP-based dynamic path planning and energy optimization framework. The discussion integrates the key findings from the simulation results, analyzes the trade-offs observed in comparison with representative baseline methods, and outlines open research questions and future research directions. Practical managerial significance is also discussed to demonstrate the applicability of the proposed approach in real-world wireless sensor network deployments.

### 5.1. Discussion of Key Findings

The simulation results demonstrate that the proposed framework achieves a favorable balance among data collection delay, energy efficiency, and network lifetime. By jointly optimizing discrete rendezvous point visiting sequences and continuous dwell-time allocation through a unified MILP formulation, the proposed method effectively reduces unnecessary mobile sink movement while maintaining controlled energy consumption across sensor nodes.

Compared with heuristic and evolutionary baseline approaches, the proposed method consistently exhibits lower data collection delay. This improvement can be attributed to the rendezvous point-based clustering mechanism and the global optimization of the mobile sink trajectory, which together reduce redundant traversal and minimize queuing latency at data collection points. Although certain baseline methods may achieve comparable or slightly better performance in isolated metrics under specific conditions, they generally lack a global optimization mechanism and therefore exhibit less predictable delay behavior or inferior energy balancing over long-term operation.

Furthermore, the periodic invocation strategy for the MILP solver significantly reduces computational overhead, achieving over 98% runtime reduction compared to full per-round optimization, while preserving the performance benefits of global path planning. This confirms that the proposed framework is not only effective but also computationally feasible for long-term network operation.

### 5.2. Open Research Questions and Future Research Directions

Despite the promising results, several open research questions (ORQs) remain and provide directions for future work:

ORQ1: Scalability of MILP-based optimization in large-scale networks. While periodic MILP invocation substantially improves computational efficiency, further research is needed to investigate decomposition or hierarchical optimization strategies for very large-scale networks with hundreds or thousands of sensor nodes.

ORQ2: Adaptive parameter tuning under dynamic environments. In the current framework, weighting parameters such as the energy decay factor and dwell-time cost are predefined. Future research could explore adaptive or learning-based mechanisms to dynamically adjust these parameters in response to changing traffic patterns, node failures, or energy heterogeneity.

ORQ3: Robustness under realistic operational uncertainties. Future studies may incorporate stochastic elements, such as variable mobile sink speed, packet loss, or intermittent communication failures, in order to further enhance the robustness of the proposed framework in real-world deployments.

These research directions aim to extend the applicability of the proposed approach while preserving its core advantage of joint trajectory and dwell-time optimization.

### 5.3. Practical Managerial Significance (PMS)

From a practical and managerial perspective, the proposed framework is well suited for time-sensitive wireless sensor network applications such as smart agriculture, industrial monitoring, and environmental surveillance. In a representative smart agriculture scenario with distributed sensor nodes over a large field, reducing average data collection delay directly translates into faster detection of critical events such as soil moisture deficiency or temperature anomalies.

Moreover, the significant reduction in optimization runtime achieved through periodic MILP invocation enables deployment on resource-constrained edge servers, making centralized or semi-centralized network management feasible without excessive computational burden. Network operators can benefit from predictable delay performance and improved energy balancing, which simplifies maintenance planning and extends operational lifetime without frequent manual intervention.

Overall, the proposed framework provides actionable decision support for practitioners by offering a systematic approach to mobile sink scheduling that balances performance efficiency with operational feasibility.

## 6. Conclusions

This paper formulated a new dynamic path planning strategy by using MILP, which shows significant performance advantages in delay-sensitive wireless sensor networks. Its balanced network lifetime management, low latency, and efficient path planning can provide innovative solutions for energy optimization and real-time requirements. However, there are still problems such as high computational complexity and insufficient adaptability to large-scale networks for MILP. In the future work, it is necessary to further explore the combination of heuristic algorithms or constraint relaxation strategies to reduce the complexity of the solution and validate the robustness of the algorithm to dynamic interference and node failure in real WSN scenarios. Meanwhile, deepen the multi-objective collaborative optimization model of path planning, energy consumption, and data quality, and then expand the application potential of the algorithm in complex heterogeneous environments. Our study provides theoretical support and technical reference for dynamic path planning and energy management and lays a practical foundation for future research.

## Figures and Tables

**Table 1:** Computational overhead comparison.

Strategy	MILP Calls	Total MILP Runtime (s)	Runtime Reduction
Full re-optimization	10,000	144.12	–
Proposed periodic strategy	110	1.80	98.75%

**Table 2:** Parameter settings.

Parameters	Value
Simulation area	100 m × 100 m
Number of nodes (N)	100
Initial energy ($E_{0}$)	1.5 J
Energy consumption per unit bit for transmission or reception ($E_{elec}$)	50 nJ/bit
Fusion unit bit energy consumption ($E_{DA}$)	5 nJ/bit
Free-space model magnification coefficient ($\varepsilon_{fs}$)	10 pJ/bit/m^2^
Amplification coefficient of bidirectional fading model ($\varepsilon_{mp}$)	0.0013 pJ/bit/m^4^
Data packet size	4000 bit
Mobile sink speed (V)	10 m/s
Communication range ($R_{C}$)	50 m
Path length penalty weight (γ)	0.3
Spatio-temporal correlation parameter (α)	0.1
Residence time weight (β)	0.5
Time-correlation parameter ($\lambda_{time}$)	5

**Table 3:** Performance comparison between the proposed MILP and state-of-the-art methods.

Performance Indicators	H-LEACH/WOA	SOGA/AESA	MILP (Ours)
FND (round)	>1000 ± 0	>1000 ± 0	319.90 ± 49.44
HND (round)	>1000 ± 0	>1000 ± 0	763.20 ± 46.46
Average delay (s)	0.30 ± 0.05	0.14 ± 0.01	0.05 ± 0.01
Average path length (m)	500 ± 50	500 ± 50	187.70 ± 77.25
Energy consumption	0.40 ± 0.02	0.29 ± 0.01	0.50 ± 0.03
Suitable application scenario	Energy-constrained	High-density network	Delay-sensitive

**Table 4:** Parameter sensitivity analysis results.

α	β	FND (Round)	HND (Round)	Path Length (m)	Delay (s)
0.05	0.30	338.00	768.00	238.99	0.01
0.10	0.30	338.00	774.00	244.02	0.01
0.15	0.30	338.00	774.00	53.56	0.14
0.20	0.30	338.00	771.00	35.46	0.09
0.25	0.30	325.00	762.00	35.42	0.06

## Data Availability

Additional data will be provided upon request.
